# Improvement in health and empowerment of families as a result of watershed management in a tribal area in India - a qualitative study

**DOI:** 10.1186/1472-698X-13-42

**Published:** 2013-10-12

**Authors:** Sandeep S Nerkar, Ashok J Tamhankar, Eva Johansson, Cecilia Stålsby Lundborg

**Affiliations:** 1 Global Health (IHCAR), Department of Public Health Sciences, Karolinska Institutet, Tomtebodavägen 18 A, SE 171 77, Stockholm, Sweden; 2 Indian Initiative for Management of Antibiotic Resistance, Department of Environmental Medicine, R. D. Gardi Medical College, Ujjain 456 006, India; 3 N.G. Acharya and D.K. Marathe College, Chembur, Mumbai 400 071, India; 4 Nordic School of Public Health, Gothenburg, Sweden

**Keywords:** Public health, Watershed management, Perceptions, Tribal, Empowerment

## Abstract

**Background:**

Tribal people in India, as in other parts of the world, reside mostly in forests and/or hilly terrains. Water scarcity and health problems related to it are their prime concern. Watershed management can contribute to resolve their health related problems and can put them on a path of socio-economic development. Integrated management of land, water and biomass resources within a watershed, i.e. in an area or a region which contributes rainfall water to a river or lake, is referred to as watershed management. Watershed management includes soil and water conservation to create water resources, management of drinking water, improving hygiene and sanitation, plantation of trees, improving agriculture, formation of self-help groups and proper utilisation and management of available natural resources. For successful implementation of such a solution, understanding of perceptions of the tribal community members with regard to public health and socioeconomic implications of watershed management is essential.

**Methods:**

A qualitative study with six focus group discussions (FGDs), three each separately for men and women, was conducted among tribal community members of the Maharashtra state of India. The data collected from the FGDs were analyzed using manifest and latent content analysis.

**Results:**

“Improvement in health and empowerment of families as a result of watershed management” was identified as the main theme. Participants perceived that their health problems and socio-economic development are directly and/or indirectly dependent upon water availability. They further perceived that watershed management could directly or indirectly result in reduction of their public health related challenges like waterborne diseases, seasonal migration, alcoholism, intimate partner violence, as well as drudgery of women and may enhance overall empowerment of families through agricultural development.

**Conclusions:**

Tribal people perceived that water scarcity is the main reason for their physical, mental and social health problems and a major obstacle for their overall development. The perceptions of tribal participants indicate that infectious diseases, migration, alcoholism, intimate partner violence and drudgery of women are end results of water scarcity and efforts to increase water availability through watershed management may help them to achieve their right to health which is embedded in their right to access to water.

## Background

The health and socioeconomic development of a community, to a major extent, depends on the availability, quality and management of its water resources. If water scarcity is the impediment in improving health and socioeconomic status of a society, watershed management can contribute to solve the problem. Integrated management of land, water and biomass resources within a watershed, i.e. in an area or a region which contributes to supply rainfall water to a river or lake, is referred to as watershed management [1,2]. The major components of watershed management include soil and water conservation to create water resources, management of drinking water, improving hygiene and sanitation, plantation of trees, improving agriculture, formation of self-help groups and proper utilization and management of available natural resources [2,3].

In most parts of the world, particularly in low and middle income countries, including India, tribal people live mainly in forests and/or hilly regions, where there is often scarcity of water, which results in sub-optimal health and socio-economic indicators [4]. Billions of people, including tribal people, do not enjoy the fundamental right to water, which is accessible, safe, acceptable and affordable. The right to water is integral to right to health according to international treaties [5]. Tribal people have to face water scarcity in many parts of the world, which suggests that their right to water and thus also right to health is not respected, protected or fulfilled. Watershed management can facilitate access to right to water, which is a fundamental element of the right to health.

Watershed management with a focus on improving sanitation/hygiene, agriculture, and social aspects can potentially contribute to improve their health along with minimizing other water related problems [1,3]. To our knowledge, scientific publications are not available on public health implications of watershed management, and research is needed to increase our understanding of how public health, watershed management, and watershed management associated socio-economic development are interlinked [3]. Qualitative studies can help to explore knowledge areas, where not much earlier research has been conducted; as such inductive studies give direct access to perceptions on the phenomenon, in this case public health implications of watershed management. Further, such studies add context to the theories, and produce a factual description of a particular issue in depth and detail [6].

The aim of this study was to increase our understanding of perceptions of tribal populations on the public health implications of watershed management.

## Methods

### Design, study setting and participants

A qualitative study with an inductive approach, using focus group discussions (FGDs) for data collection, was conducted in a tribal area of the Thane district, located north of Mumbai in Maharashtra state of India. This area is hilly with sparse and patchy forest cover and is characterized by scarcity of water during a large part of the year, although the annual rainfall is above national average, suggesting a need for conservation and proper management of water in this area. The literacy rate of the study area is 44.8% which is far below the national average (74.1%) [7]. The people in this area are dependent on the government for health services through ASHA (Accredited Social Health Activist), sub-centers and primary health centers and a community hospital in a nearby town [8]. Traditional healers, so called ‘Bhagat’ are also present. Water related diseases like diarrhoea and malaria are common in the study area.

For this study, three villages were purposively selected based on different levels of implementation of watershed management programme (Table 1). The selected villages were within a radius of 15 km and people from each village knew developmental activities in surrounding villages. In village one, (hereafter referred to as watershed management village or WM village) the watershed management programme was initiated about 10 years ago and included water conservation measures, management of drinking water, plantation of fruit trees, improved agriculture, creation of self help groups, promotion of good sanitation practices, toilet construction and other related activities. In village two, (hereafter referred to as partial watershed management village or PWM village) watershed management programme is in the implementation phase since two years and the programme includes initial stages of water conservation measures, plantation, improved agriculture, creation of self help groups, toilet construction and other related activities. In the third village, (hereafter referred to as no watershed management village or NWM village) there has so far been no watershed management activity.

**Table 1:** Background information about the study villages

**Particulars**	**Watershed management village**	**Partial watershed management village**	**No watershed management village**
Watershed management	Since 10 years	Since 02 years	No watershed development
No. of households	95	47	52
No. of dug wells	5	3	2
No. of bore wells	0	1	0
School	Up to 4th Std.	Up to 7th Std.	Up to 4th Std.
Major crops	Nagali, Rice, Mango and Cashew	Nagali, Rice, Mango and Cashew	Nagali and Rice
Distance from nearest town (Km)	15	12	18
Distance from Community Hospital (Km)	15	12	18
Distance from PHC (Km)	11	10	18
Distance from PHC Sub-centre (Km)	2	1	5

Nagali- Local cereal crop (*Eleusine species*), PHC – Primary Healthcare Centre.

### Data collection procedure

For conducting the FGDs, which were used for data collection, purposive sampling was used to select the individual participants from each village in order to achieve variation with respect to sex, age and literacy (Table 2). All participants were 18 years or above in age. An FGD guide was prepared with open ended introductory questions for the different areas to be explored (see the ‘FGD guide, showing the introductory questions’ section). The guide was piloted before use. A total of six FGDs were conducted in the three selected villages, two FGDs per village, one for men and one for women. The first author was the moderator of all the FGDs and an additional female moderator was present for the women’s FGDs. Both moderators have good understanding of customs, traditions and the local language “Marathi”. All the FGDs were conducted in Marathi and each discussion lasted for 50 to 70 minutes. The number of participants for each discussion was in between 6 to 10. The FGDs were conducted at places and times convenient for participants as well as moderators. All the FGDs were recorded using a digital voice recorder and an observer was present for note taking.

**Table 2:** Background information of participants

**Participant’s background**	**Number of participants in FGDs**					
	**Watershed management village**		**Partial watershed management village**		**No watershed management village**	
**Gender**	Women	Men	Women	Men	Women	Men
**Age**						
**18-25 yrs**	3	1	2	1	2	1
**26-59 yrs**	5	6	5	4	4	5
**>59 yrs**	1	2	1	1	1	0
**Education**						
**Illiterate**	6	4	4	2	6	3
**Primary**	1	2	2	2	1	1
**Secondary**	2	2	2	1	0	1
**Higher**	0	1	0	1	0	1

Illiterate- Not able to read or write, Primary- Std. 1 to Std. 4,

Secondary- Std. 5 to Std. 10, Higher- Std. 10 and above.

### FGD guide, showing the introductory questions

Introductory question

What is the situation of drinking water for the community in the village?

What kind of crops do you grow for home consumption and sale?

What are the hygiene and sanitary practices followed by villagers?

What is the situation of water related diseases in your village?

How do you tackle your health problems?

Do you have any idea about watershed management?

(Explanation about watershed management if needed)

How can watershed management activities influence your health?

Anything to add

### Data management and analysis

The recorded FGDs were transcribed verbatim in Marathi and translated into English by the first author (SSN). These translations were cross checked by one of the co-authors (AJT), having command over both the languages. The first and second authors have a good understanding and long experience of participating in projects in rural India. All authors have a public health perspective and experience from qualitative research and varied professional backgrounds viz. agriculture, environmental health, nursing and pharmacy.

Manifest and latent content analysis was used as methods for analysis [9]. First, open codes were identified in the text. Both transcripts *viz*. English and Marathi were used while identifying codes to understand the actual meaning of the text. These codes were grouped into sub-categories according to similar patterns and sub-categories were further grouped to form categories to achieve the manifest level of the content analysis. Finally, one theme, the underlying meaning of the text, was identified.

### Ethical considerations

This study was approved by the Institutional Ethics Committee of R. D. Gardi Medical College, Ujjain, India and informed consent was obtained from all participants as well as from local authorities. Participation was voluntary and the participants were informed that they could withdraw at any time without any implications.

## Results

The findings of the study are presented under the main theme, “Improvement in health and empowerment of families as a result of watershed management” (Table 3). Five categories are described from the manifest content analysis (i) Water centric development, (ii) Direct and indirect impact of seasonal agriculture on physical, mental, and social health, (iii) Water resource and its association with water related health problems, (iv) Effects of lack of education and meager income on utilization of health facilities and (v) Women’s role in family and social health. Differences in perceptions of the participants from the three villages in relation to twelve different codes of importance for public health have been identified in Table 4. Quotes from different FGDs are presented under each category to illustrate the findings. Within the quotes explanations by the authors are given in square brackets.

**Table 3:** Theme: Improvement in health and empowerment of families through watershed management

**Theme**	**Improvement in health and empowerment of families through watershed management**				
Categories	Water centric development	Impact of seasonal agriculture on mental, physical and social health	Water resource and its association with water related health problems	Effects of lack of education, meager income on utilization of health facilities	Women’s role in family and social health
Sub-categories	Socio-economic development	Unemployment leads to frustration, alcoholism	Impact of water availability on sanitation and hygiene	Access to healthcare	Drudgery of women
	Water resource development	Impact of migration on physical, mental and social health	Management of drinking water	Misconception due to lack of knowledge	Women empowerment
	Agricultural development				

**Table 4:** Differences perceived by participants on particular codes of importance to public health in their village

**Sr. No.**	**Codes**	**Watershed management village**	**Partial watershed management village**	**Non watershed management village**
**1**	Water availability	Improved up to certain extent	Improving and hope for further improvement	Water scarcity for 5–6 months, no difference
**2**	Agriculture, income and food	Change in cropping pattern, plantation crops, vegetable crops, income level increasing, improving nutrition	Changing cropping pattern, plantation crops, floriculture, income level increasing, improving nutrition	Rain-fed agriculture, no change in income and nutrition
**3**	Migration	Migration reduced, mostly men migrated without family for shorter period	Migration reduced, mostly men migrated without family for shorter period	Migration with family is must for most of the families
**4**	Education level	Increased awareness about children education, girl’s education	Increased awareness about children education, girl’s education	Education is of secondary importance
**5**	Hygiene and sanitation	Hand washing in practice, fewer number of toilets in use	Hand washing in practice, use of toilets in each house	Less use of water for hygiene and sanitation purpose, no toilets, hand washing mixed opinions
**6**	Epidemic diseases	Almost no epidemic in last decade but few diarrhoeal cases in rainy seasons	Very low	Diarrhoeal diseases regular in rainy season but under control
**7**	Self-help groups	More number of groups exist and functioning	Exist and functioning	Tried but failed
**8**	Domestic violence	Comparatively less	Comparatively less	Comparatively more
**9**	Hard work of women	Reduced and comparatively less	Comparatively less, still burden of water collection	More burden on women for water and firewood collection
**10**	Alcoholism in men	Exists but reduced and under control	Very low	Exists, troublesome to women and society and not under control
**11**	Availability of firewood	Increased	Hope to generate in own field	No difference, extra burden of work on women
**12**	Utilization of health service, modern medicine	More active and time conscious	More active	Comparatively less active, opt for traditional measures and have income and infrastructural obstacles

### Water centric development

Participants from all the FGDs were of the opinion that water scarcity is the major problem in the area. Their perceptions of the severity of the problem varied from village to village, as every village had different levels of water availability. In the WM and PWM villages, agriculture had relatively prospered compared to NWM village and villagers in the WM village reported benefits in terms of economic gain. The participants from PWM village perceived, that watershed management was beneficial for agriculture as it retains moisture, which could be used to grow an additional or a new crop. This was felt as an empowerment through generation of income as well as employment.

Fruit crops plantation locally referred to as ‘Wadi’ meaning ‘small orchard of fruit crops’ enabled through watershed management was perceived as a hope for sustainable development in all FGDs. Vegetable production was also perceived as a potential area for increase in income as well as for increasing the nutritional status. People from PWM village could clearly remember the benefits of watershed management, as it was a recent experience.

“Here our land is on hilly terrain. [Generally] there is scarcity of water.....if we wanted to do something different; there was a problem of water...... There are lots of benefits of watershed management. We can [now] sow beans. Even if rain stops, [fields at downside retain moisture] we can take those crops.” (FGD-Men, PWM village)

The participants from WM village perceived, that plantation of trees across the slopes and contour trenches had helped to improve water conservation and it had also resulted in improvement of the quality and quantity of water in the well. Additional benefits of trees were summed up as,

“....Tree shade gives protection from heat. Humans and cattle can take rest underneath the trees....trees are used as firewood for cooking purposes, and also to burn wood and dry leaves [are used] at nursery sites.....the wood from trees can be used [in kiln] for making bricks and to construct houses.”(FGD-Men, WM village)

Women participants from the NWM village emphasized that due to scarcity of water, development in their village had stopped. Here, women seemed to lack their right to access to water and they mentioned its importance to them. They had however hopes that if water would become available that would make a difference as they will be able to grow vegetables and fruit crops. They also had a feeling that if water was easily available, they could save time now spent in the collection of water, for other more productive activities.

“If we have enough water, we will grow vegetable crops. We will grow crops in a better way and we may stay on the farm itself.....we should get water near our house....we should get clean [drinking] water....we also need ‘Wadi’ [small fruit crop orchard].” (FGD-Women, NWM village)

Some participants also had an interesting observation, that in the beginning of the implementation of the watershed management programme, the water level increased, but after the plantation of *Eucalyptus* [locally called Nilgiri] trees, they became the reason for ground water depletion. Participants from WM village viewed, that due to this phenomenon, many villagers had a misconception, that all trees absorb more water as they grow and therefore become the reason for water depletion. However participants from WM village did not blame cashew and mango trees for such an effect.

### Direct and indirect impact of agriculture on physical, mental, and social health

Participants from NWM village informed, that their agriculture is dependent on rains and their crops are meant mostly for household consumption and not for sale. They also perceived that their narrow spectrum of crops and low productivity results in insufficient amount of food for their families. Participants from WM village perceived that the physical look of a person is an indication of his or her nutritional status, which they felt was comparatively better in their village compared to other villages.

“A person from a neighbouring village doesn’t look good physically, these much of cheeks [showing by acting] are inside.” (FGD-Men, WM village)

All groups perceived that poverty and unemployment in the area are results of rain fed agriculture. The participants from NWM village viewed, that unemployment forced them into seasonal migration in search of jobs, such as road or house construction, farm work, etc. They informed that, the earnings received through wages after migration was the main source of income to run their families. The need for migration to earn some money for sustenance of their family, made them feel powerless.

“For money, we have to go somewhere…what can we do if we don’t have money. If we are at home, we do not get money. We have to go outside [away from the village].” (FGD-Men, NWM village)

In the WM village and PWM village, participants perceived that migration had been reduced drastically after plantation of fruit crops. In NWM village, women informed that to migrate with the whole family to other location for longer period was necessary for them to earn some money.

“Migration has decreased. Due to Wadi, one man is completely engaged. It decreased [migration] very much. When Wadi is planted, we cannot go outside.” (FGD-Men, WM village)

Women participants from NWM village strongly felt that they do not receive respect at the migrated sites and have to face social disparity problems. Thus they reported feeling uncomfortable at migrated sites. Unwilling migration and social disrespect were looked upon with anger by the women and it disturbed their peace of mind. Their feeling of not having the basic rights like access to water and sanitation, respect to their dignity, in addition to feeling powerless and socially underprivileged became even stronger in these situations. Participants from NWM village also perceived, that they were more prone to communicable diseases, such as malaria at migrated sites, as the employer usually did not arrange proper accommodation. They reported that most of the time, they had to live in open places, where mosquitoes are abundant and mosquito biting is high. In addition to that participants mentioned that they do not get proper health services at the [migrated] sites, as they do not belong to that place.

“*Live anywhere, eat anything. Who is going to take care? We have to do this as we have to fill our stomach. Do whatever job we get to earn some money......We have to live in open places. There are too many mosquitoes there. They bite us during the whole night. We cannot have a comfortable sleep. It causes shivering fever and we do not get proper health services there, being an outsider.” (FGD-Women, NWM village)*

There were mixed views about unemployment or absence of work and its relation to alcoholism in other villages, but in NWM village women participants viewed that non-availability of water resulted in men having no work to do in the village as there was no agriculture or growing of fruit crops and that this absence of work was one of the main reasons for alcoholism in men. According to them, absence of work means no income which led men to frustration and alcohol addiction. Women participants also mentioned that alcoholism not only affects men’s health, but also disturbs their family life and incidences of intimate partner violence occurred mostly due to alcoholism. Alcoholism was perceived as an important health issue by both men and women in NWM village. This was not reported in WM village or PWM village.

“Earlier it [alcoholism] was more. Now it is stopped. There was no work here. Now Wadis have been grown. Crops of cashew, mango are grown. ....” (FGD-Men, PWM village)

When asked about sexually transmitted diseases or HIV/AIDS, most participants seemed to be ignorant.

### Water resource and its association with water related health problems

Participants said that they had to change their source of drinking water in the summer season, for few days for WM & PWM villages and for several months in NWM village, because of insufficient water in the nearest source. According to participants from all FGDs and especially from NWM village, if they get assured supply of water from a single source throughout the year, then disinfection of water (destruction or prevention of growth of microorganisms for example through chlorination) can be done regularly and diarrhoeal diseases can be reduced. Awareness regarding disinfection of drinking water suggests earlier experience of epidemics of water borne diseases in all villages.

“If we get it [water] for 12 months from the same well, then illness (diarrohea) will not be much as water will get disinfected regularly.” (FGD-Men, NWM village)

All the participants informed that water scarcity affects the sanitation practices as well as personal hygiene. Open air defecation is the practice irrespective of the owning of a toilet. In general, participants in all FGDs were of the view that use of toilets varies dramatically from person to person, season to season and depends on the availability of water. Women participants did not consider hygiene at household level as one of the factors for diarrhoeal and other diseases but thought it was community resource originated.

In NWM village, both men and women participants viewed that they do not have toilets as it is not useful for them because of water scarcity so they follow open air defecation. In comparison, most participants from WM and PWM villages, perceived use of toilets as hygienic and they used them as well.

### Effects of lack of education, meager income on utilization of health service

Participants from WM and PWM villages perceived education as an important tool for advancement in life as well as for obtaining better health service and they felt that even though they were not much educated, their children were becoming educated due to income enhancement as a result of the implementation of watershed management programme and that will make a difference in the future. Education was felt to be a part of empowerment in social health whereby people gain knowledge and confidence in ordinary life. They also viewed, that if some people change their behavior, other community members will follow, so if people follow educated people in society, it can bring a positive change in society.

“Now our children become educated… there is a difference due to education.....If we behave good, then other person also tries to behave good. One follows other.” (FGD-Men, PWM village)

Both the men and the women participants in the study perceived that the traditional healers, the ‘Bhagats’ in their area, have an important role in the health service as they are either the first or the last solution for their illness as people have faith in them. Sometimes, the ‘Bhagat’ is the person, who recommends them to go to community hospital for modern medicine. Participants from WM village felt that, lack of awareness and low educational level in their area implies that it will take time to change the attitudes of the people towards health service.

“In the beginning, we take tablets, medicines. If not cured, then we go to Jawhar or Mokhada [towns]…at the community hospital…if not cured, then we will keep treatment of hospital and will also show to Bhagat.” (FGD-Men, NWM village)

Participants viewed that, income level also determines the utility of public health service, as people have to have money and time to travel to a town place or a centralized place where facilities have been created. Sometimes they have to buy medicines from a private medical shop, because of non-availability of particular medicines at the public hospital, which again was an extra economic burden. People from WM village expressed that few of them have started to go to private practitioners, which is more costly, and that they are also paying for medicines from the private medical shop as now they can afford it.

### Women’s role in family and social health

In NWM village, women participants perceived that they work harder than men.

“Men do not work much. Only women work hard. Whether men can do the work of women? Work on farm, taking care of children and family, maintenance of house by dung slurry! After finishing the work at home, we have to follow the men for farm work.” (FGD-Women, NWM village)

Women in WM village thought that the work done by men and women in their village was equally hard. Men participants from WM village and PWM village admired the hard work of women.

Women participants informed that they had to carry water and firewood from a long distance. They felt that it is a hard work, which made them tired and they had to spend most of the time for this type of work. The women participants also said that their daughters also have to do the same work. Education is second priority for girls after household work. The women participants from WM village informed that, the hard work of bringing firewood from a long distance had been reduced after plantation of the trees in the process of afforestation in their village during the watershed management programme. So it was possible for their daughters to attend school.

“We [used to] carry it [firewood] on head....those who have trees in their village, they do not go now.....All were going [to collect firewood], when trees were small....now trees are grown up, so few are going. We were bringing it from a long distance. If we leave at 5 am then, [we would] return back in evening at 5 pm. We became very tired after that.” (FGD-Women, WM village)

Men participants from WM and PWM villages acknowledged the time and hard work that women spent for collection of water and viewed that the time saved could be utilized for other productive work or education. They also viewed that women play a major role in the control of water borne diseases by supervising disinfection of water in the well. Women participants also informed that they do most of the work related to hygiene and sanitation at household level, emphasizing their role.

“Woman does it alone. Man does not. Carry it [a pot with water] on head,.... All washing pots, clothes, [working with] farm yard manure, cooking food, etc. are done by woman only.” (FGD-Women, WM village)

Apart from work, women also viewed that they had to suffer from intimate partner violence due to alcoholism in men. Social activities such as formation of self-help group by women and their participation in them were viewed as a positive development taken place due to watershed management. According to the women, it gave them some kind of empowerment and helped them to improve social health by helping in controlling unsocial things, like alcoholism.

## Discussion

This study reflects perceptions of a tribal community on implications of watershed management for public health. According to the perceptions of the participants, agricultural and socio-economic development, education and awareness, hygiene and sanitation and improved water availability resulting from the implementation of an integrated watershed management programme have a positive impact on physical, mental and social health of the people. Effective use of available water for household and agricultural purpose was viewed as a key factor for improvement in health through safe drinking water, proper nutrition and increase in income level. The perceptions of participants suggest that watershed management might be a good approach to empower tribal families with enhanced health status by increasing their income level, educational opportunities and by building their self confidence for the sustenance of life. The results of our study and analysis suggest that the tribal people, who participated in our study, perceived watershed management programme as empowering them to claim their right to water and right to health.

Participants perceived that scarcity of water is a major constraint in the overall development of their villages, which limits their capabilities for obtaining health benefits. It affected their income level and socio-economic development. These findings are consistent with a model developed on water and economic growth, where it is suggested that economic growth is positively associated with effective utilization of water resources [10]. An earlier study from India has informed on importance of socioeconomic status in reducing population-level health disparities in the society [11]. Our study indicates that socio-economic development achieved by overcoming water scarcity through watershed management, can result in improved public health for the society.

In India, indicators of agricultural performance or income level have a strong relationship with indices of under-nutrition among children and adults [12]. The view of our study participants about low productivity in agriculture and subsequent malnutrition are in accordance with this.

The selection of tree species for plantation, depending upon the type of soil and average rainfall, in a particular area, is very important as it decides the fate of ground water [13]. A majority of the participants perceived that plantation of trees across the slopes, a part of watershed management programme, helps to stop soil erosion and also improves ground water table. Some participants also perceived that *Eucalyptus* tree plantation reduces ground water table, which has been shown to be true at least in an African setting [14]. The mention of association of *Eucalyptus* trees with depletion of ground water shows the keen observation of the tribal people of their natural surroundings.

Seasonal agriculture results in no work in other seasons, which forces tribal people to migrate in search of employment, a migration which often leads to various health problems/challenges. Earlier studies present similar views in relation to health issues of migrants and seasonal farm workers. For example, work related health problems and chronic illness [15] and poor quality of accommodation, geographical isolation, crowding, deficient toilets and poor cooking facilities were identified as determinants of poor health under such conditions [16]. Also, according to Borre, et al. [17], food insecurity, poverty and dependency is rooted in the cultural lifestyle of farm work. Women participants viewed that, sometimes they are treated as being in a lower class in social structure when they migrate, which makes them feel uncomfortable and suppressed mentally. This is consistent with the findings of Kim-Godwin and Bechtel [18], who inform that 51 percent of the migrants perceived themselves being under stress which put them at a greater risk of experiencing psychological difficulties. Participants view on transmission of communicable diseases like malaria during migration is consistent with the findings of Saxena and Devadethan [19], who report that seasonal migration of labour has impact on the spread of malaria. Similar results are reported from studies conducted in rural Venezuela [20], Northern Thailand [21] and Rajasthan, India [22]. Though in our study, participants were not much aware of HIV/AIDS, a study on migration and HIV epidemic in south west India suggests that there is an increased risk of spread of HIV infection during seasonal migration [23]. Due to lack of awareness and illiteracy, tribal people may even be at a greater risk of getting HIV infection during migration. However, migration along with families, as reported in our study, may be a factor that restricts the spread of sexually transmitted infections and hence the participants were unaware of HIV/AIDS infections. In view of our participants, watershed management can overcome the problem of seasonal agriculture and subsequent migration by effective utilization of available water resource to increase the productivity, thus indirectly helping to solve public health challenges associated with migration.

All the women participants perceived that improper sanitation practices like open air defecation are due to water scarcity. One important aspect mentioned by participants is that if they have one assured drinking water source all the time, it is more likely that it will get disinfected regularly, thus reducing the occurrence of diarrhoeal diseases associated with water. This shows that watershed management plays a role in the reduction of water borne infectious diseases like diarrhoeal epidemics and results in improved public health status. Thus, it can be said that access to water is inter-related to the right to health of tribal people. In India, about 25 percent of villages do not have assured drinking water source for about four to five months per year [24].

Interestingly, hygiene at household level was not perceived by women participants as a possible reason for contamination of household drinking water. This is in contrast with earlier research, which suggests that microbiological quality of drinking water declines from source to point-of-use [25].

In general, participants said that they follow superstitious or traditional health measures like going to ‘Bhagat’ because of lack of education, poor infrastructural health facilities, their income level, time constraint, etc. However, participants from WM village informed that some of them can now afford to go to private practitioners at town place which indicates that the slight increase in their income level resulting from watershed management allows them to spend more on health.

In all FGDs, the perception of all participants was that, in the tribal community, women play a key role in the functioning of the family. Similar view has been put forward by an earlier study in India [26]. Unless women are involved in the decision making process, long term sustainability of developmental efforts cannot be achieved [27]. It appears that if women are involved and play a central role in the watershed management programme, it will help in attaining improvement in social health.

In watershed management villages, men and women showed respect to each other’s work. The respect shown to opposite genders’ work might be a step towards more gender equality brought about by watershed management.

The perceptions of women participants suggest that plantation of trees associated with the watershed management programme saves their time and energy spent in the collection of firewood. This indicates that the development work like plantation not only helps to protect natural environment by rejuvenating resources but also reduces the hard work of women in tribal areas. These perceptions of women participants are supported by the study of Singh et al. [28] which states that in majority of the states of India the workload of women is reduced up to one to two hours daily as an impact of watershed management. According to the FAO [29], along with poor diet, heavy workload weakens women’s health. A reduction in hard work as a result of watershed management may thus be helpful in the improvement of women’s health.

Education was also perceived as a domain of empowerment by women participants. Importance given to their daughter’s education is a reflection about their awareness about education. Time saved by women and girls as a result of developmental work in watershed management programme can be spent on educational activities which makes them feel empowered.

Drawing from earlier experiences, the Government of India formulated common guidelines for watershed development projects in 2008, in which formation of self-help groups (SHGs) is recommended [30]. Women from all study villages found SHGs useful to unite at a place regularly as it gives them a platform to discuss issues of importance to them and find solutions for them. In tribal areas, union of women at village level can solve the social issues like alcoholism. Women participants found it as a key of empowerment to them. Thus, through education and awareness, watershed management helps in the empowerment of women which is a key rights issue. This empowerment will in turn lead to women being able to claim their rights in various fields leading to improved psychological, social and physical health.

Various studies support the link between alcoholism and intimate partner violence in different parts of the world [31,32]. Jaysuriya et al. [31] reported that in Sri-Lanka, young women are at increased risk of violence from their alcohol addicted partners. Study participant’s perceptions suggest that the intensity of alcohol addiction varied in men according to the watershed development there and at the same time women’s reaction on it also varied accordingly in respective villages. This suggests that social issues are associated with development in that locality which facilitates women in handling the unwanted situations. Earlier research supports the views among our participants regarding the relationship between socio-economic development, alcoholism in men and intimate partner violence [26,27]. Watershed management might be a pathway in the improvement of social health through socio-economic development of tribal community.

From the foregoing, it appears that tribal participants perceive that implementation of a watershed management programme has a positive impact on both public health and socioeconomic development and the improved socio-economy resulting from watershed management has a further augmentative effect on public health. Parkes et al. [3] pointed out that research is needed to improve our understanding of how public health, wellness and watershed-based management fit together. We hope that this paper makes such a contribution.

Our findings suggest that public health problems in tribal areas are complex and several of them are directly and/or indirectly associated with water availability. As far as the potential of watershed management in tribal areas is concerned, we suggest developing an area - geographical area - specific health policy that has an integrated water, health, agriculture and socio-economic management approach. In this context, the definition of ‘area’ is, ‘a village or group of villages with similar agro-ecosystem that come under one watershed’.

We propose that as a precursor for facilitating their right to health and well being, people should identify their problems and should integrate at local level to find solution to their problems. There should be a public policy that promotes the awareness among community regarding such integrated efforts and when people come forward with a solution, there should be enough support from local authorities/government to achieve the desired results.

Watershed management is integral to right to water issues, which are a fundamental element of the right to health. This paper gives us a better understanding of the impact of right to water issues on the right to health of tribal populations as we chose a qualitative research methodology that allowed us to listen to the voices of those most in need of watershed management and whose voices are often not heard. Though not exactly similar, the approach is akin to the ‘localising human rights approach’, with focus on empowerment for attaining better public health standards, a key human rights concept [33].

We suggest that with respect to the right to access to water, which has a high impact on physical, social and mental health, researchers and policy implementing states/agencies should take a ‘bottom up’ view, which will help in identifying the real problems by listening to the voices of those affected by rights deprivations.

The Government of India (2008) developed guidelines for watershed management projects that have a holistic approach. This approach needs government’s efforts as well as participation of the people to make watershed management successful so that it is a sustainable development resulting in achieving better health status. The guidelines also suggest the responsibility of the people to act, to be able to claim their rights. We propose that wherever people suffer from lack of appropriate access to water (either due to deficient rainfall or topography resulting in quick loss of rainfall water), governments all over the world should take up holistic watershed management programmes, to fulfill the rights of their people to have access to water and access to health. In this effort, peoples’ participation is a key for sustainability, as people need to develop an ownership feeling to derive best benefits.

### Methodological considerations

FGD is a technique, used in qualitative research which is open to explore anticipated as well as unanticipated issues. It requires a fairly low budget and gives relatively quick results [34]. For this study, FGD was suitable, as most of the participants were not literate and it was easier for them to express their views in group rather than in individual interviews. During the data analysis, we used data triangulation to increase the trustworthiness of the study. Cross checking of the translated data with recorded data was done to avoid misinterpretation of the data. The strength of the study is that all authors have a public health perspective, have experience in qualitative research and have varied professional backgrounds viz. agriculture, environmental health, nursing and pharmacy. The different educational, geographical and cultural backgrounds of the authors, with extensive experience in their own field of research brought a unique perspective to the study that enhanced the conformability of the results. Member check was carried out with informal discussion with study participants to find out that their perceptions were interpreted correctly. This is a qualitative study based on focus group discussions, conducted in a tribal belt of India. The results might however be of value for readers from a wide range of contexts with similar types of water scarcity.

## Conclusions

The participants from the tribal community viewed that water scarcity is the main obstacle in their socio-economic development and consequently a reason for their various physical, mental, and social health problems. They perceived that water scarcity resulted in seasonal agriculture, migration, water related infectious diseases, alcoholism and drudgery of women and watershed management and the developmental aspects associated with it was an inducer of improved public health, enhanced socio-economic status and also a cause of empowerment of the families. The overall empowerment of families and especially of women, enable them to claim their rights. Our research shows that local people’s experiences identify the rights that suffer when their right to water is not respected, e.g. the right to health, the right to education and the gender dimension of this, the rights of girls and women.

## Abbreviations

ASHA: Accredited social health activist; FAO: Food and agriculture organisation; FGD: Focus group discussion; NGO: Non-government Organisation; HIV/AIDS: Human immunodeficiency virus/acquired immune deficiency syndrome; PHC: Primary healthcare centre; SHG: Self-help group.

## Competing interest

The authors declare that they have no competing interest.

## Authors’ contributions

SSN, AJT and CSL designed the study. SSN interviewed, transcribed and translated the transcripts from Marathi to English. AJT cross checked the translations. SSN did the preliminary analysis. All authors contributed to the final analysis of the FGDs. All authors have read and approved the final manuscript.

## Pre-publication history

The pre-publication history for this paper can be accessed here:

http://www.biomedcentral.com/1472-698X/13/42/prepub
